# Managing chronic myeloid leukaemia in the elderly with intermittent imatinib treatment

**DOI:** 10.1038/bcj.2015.75

**Published:** 2015-09-18

**Authors:** D Russo, M Malagola, C Skert, V Cancelli, D Turri, P Pregno, M Bergamaschi, M Fogli, N Testoni, A De Vivo, F Castagnetti, E Pungolino, F Stagno, M Breccia, B Martino, T Intermesoli, G R Cambrin, G Nicolini, E Abruzzese, M Tiribelli, C Bigazzi, E Usala, S Russo, A Russo-Rossi, M Lunghi, M Bocchia, A D'Emilio, V Santini, M Girasoli, R Di Lorenzo, S Bernardi, A Di Palma, B M Cesana, S Soverini, G Martinelli, G Rosti, M Baccarani

**Affiliations:** 1 Unit of Blood Diseases and Stem Cell Transplantation, University of Brescia, Brescia, Italy; 2 Ematologia 1-TMO, AOR Villa Sofia-Cervello, Palermo, Italy; 3 S.C. Ematologia, Dipartimento di Oncologia ed Ematologia, A.O.U. Città della Salute e della Scienza di Torino, Torino, Italy; 4 Dipartimento di Terapie Oncologiche Integrate, IRCCS AOU S. Martino-IST, Genova, Italy; 5 Institute of Hematology 'L. & A. Seràgnoli', DIMES, University of Bologna, Bologna, Italy; 6 Division of Hematology, Department of Oncology and Hematology, Niguarda Ca' Granda Hospital, Milan, Italy; 7 Divisione Clinicizzata di Ematologia AOU Policlinico-V. Emanuele, University of Catania, Catania, Italy; 8 Azienda Policlinico Umberto I, Sapienza Università, Roma, Italy; 9 Hematology Unit, ‘Bianchi-Melacrino-Morelli' Hospital, Reggio Calabria, Italy; 10 Hematology and Bone Marrow Transplant Unit, Azienda Ospedaliera Papa Giovanni XXIII, Bergamo, Italy; 11 University of Turin, San Luigi Gonzaga Hospital, Turin, Italy; 12 Hematology and Hematopoietic Stem Cell Transplant Center, San Salvatore Hospital, Pesaro, Italy; 13 Hematology, S Eugenio Hospital Tor Vergata University, Rome, Italy; 14 Division of Hematology and BMT, Azienda Ospedaliero—Universitaria di Udine, Udine, Italy; 15 Hematology, Mazzoni Hospital, Ascoli Piceno, Italy; 16 U O Ematologia e CTMO Ospedale A., Businco-Cagliari, Italy; 17 UOC Ematologia AOU 'G Martino' Policlinico Universitario di Messina, Messina, Italy; 18 Division of Hematology, University of Bari, Bari, Italy; 19 Division of Hematology, Department of Clinical and Experimental Medicine, Amedeo Avogadro University of Eastern Piedmont, Novara, Italy; 20 Hematology and Transplants, University of Siena and AOUS, Siena, Italy; 21 Department of Cellular Therapies and Haematology, San Bortolo Hospital, Vicenza, Italy; 22 Unità di Ematologia, AOU Careggi, University of Florence, Florence, Italy; 23 Hematology Department, 'A. Perrino' Hospital, Brindisi, Italy; 24 Division of Haematology, Spirito Santo Hospital, Pescara, Italy; 25 DMMT, Unit of Medical Statistics, University of Brescia, Brescia, Italy; 26 Department of Haematology-Oncology 'L. and A. Seràgnoli' – S. Orsola Malpighi Hospital, University of Bologna, Bologna, Italy

## Abstract

The aim of this study was to investigate the effects of a non-standard, intermittent imatinib treatment in elderly patients with Philadelphia-positive chronic myeloid leukaemia and to answer the question on which dose should be used once a stable optimal response has been achieved. Seventy-six patients aged ⩾65 years in optimal and stable response with ⩾2 years of standard imatinib treatment were enrolled in a study testing a regimen of intermittent imatinib (INTERIM; 1-month on and 1-month off). With a minimum follow-up of 6 years, 16/76 patients (21%) have lost complete cytogenetic response (CCyR) and major molecular response (MMR), and 16 patients (21%) have lost MMR only. All these patients were given imatinib again, the same dose, on the standard schedule and achieved again CCyR and MMR or an even deeper molecular response. The probability of remaining on INTERIM at 6 years was 48% (95% confidence interval 35–59%). Nine patients died in remission. No progressions were recorded. Side effects of continuous treatment were reduced by 50%. In optimal and stable responders, a policy of intermittent imatinib treatment is feasible, is successful in about 50% of patients and is safe, as all the patients who relapsed could be brought back to optimal response.

## Introduction

More than 80% of patients with Philadelphia-positive (Ph+), BCR-ABL1+, chronic phase chronic myeloid leukaemia (CML) are alive after >5 years and are projected to have a life expectancy very close or even identical to that of a non-leukaemic matched control population.^1, 2, 3, 4, 5^ These results were obtained using the tyrosine kinase inhibitor (TKI) imatinib (Gleevec or Glivec, Novartis Pharmaceutics), frontline.^1, 2, 3, 4, 5, 6, 7, 8, 9^ Some of these patients, in a proportion estimated to range between 20% and 40%, achieve a deep molecular response (DMR), that is to say a BCR-ABL1 transcripts level ⩽0.01% on the International Scale.^9, 10, 11, 12^ About 50% of them were reported to maintain that remission status after discontinuation of imatinib and to achieve a stable treatment-free remission (TFR).^13, 14, 15, 16^ The introduction of the so-called second-generation TKIs, both in first and second line, is expected to fare even better, with up to 50% or more of the patients achieving a DMR,^17, 18, 19, 20^ and up to ⩾50% of them entering into a TFR status. If these expectations will be fulfilled, the proportion of patients who will be in TFR will range between 25% and 50%. However, about ⩾50% of all patients will not be able to discontinue, and for them, the current policy is to continue the treatment with the same TKI, at the same dose and schedule, indefinitely and lifelong.^3, 5^ Until today, the case of the chronic treatment of these patients has not received the same attention as the case of TFR policies. But the issue is important for obvious reasons of quality of life,^21^ treatment-related side effects and complications^22, 23, 24, 25, 26, 27, 28, 29^ and also because of drug and management costs.^3, 5, 30^

For these reasons, we designed and initiated a pilot study testing the effect of a non-standard, dose-reduced, policy of imatinib treatment.^31^ We report here on the long-term results of that trial.

## Patients and methods

The study (EUDRACT protocol number 2007-005102-42, approved by the Ethic Committee of the Spedali Civili of Brescia, Italy and registered at ClinicalTrials.Gov with the number: NCT00858806) was limited to patients aged ⩾65 years who had been treated first line with imatinib once daily (OD) for chronic phase CML, for a minimum of 2 years, and were in complete cytogenetic response (CCyR). One hundred and fourteen patients were screened in 24 GIMEMA (Gruppo Italiano Malattie Ematologiche dell'Adulto) centres. Nineteen patients (17%) did not fit the inclusion criteria, 19 (17%) did not consent and 76 were enrolled. The median age at enrolment was 72 years (range 65–83 years). The median duration of imatinib treatment was 5.75 years (range 2.0–6.6 years). Sokal risk^32^ distribution at diagnosis was 33% low risk, 55% intermediate risk and 12% high risk. At enrolment, all patients were in CCyR, and all but one were in major molecular response (MMR or MR^3.0^, BCR-ABL1 transcripts level ⩽0.1% on the International Scale).

The daily dose of imatinib was not modified (400 mg OD in 81% of patients, 200–300 mg OD in 17% of patients and 600 mg in one patient), but imatinib was given 1 week on/1 week off for 1 month, 2 weeks on/2 weeks off for another 2 months and then on a 1 month on/1 month off schedule. The protocol originally mandated to proceed with the intermittent schedule as long as the CCyR was maintained so that the return to continuous daily treatment was mandatory only in case of CCyR loss. After 2 years, an amendment allowed a return to the continuous daily schedule also in case of MMR loss.

The cytogenetic response was assessed by chromosome banding analysis of marrow cell metaphases or by interphase fluorescence *in situ* hybridization analysis of peripheral blood cell nuclei, as described elsewhere.^33^ The cytogenetic test was performed every 6 months for the first 2 years, and then only in case of loss of MR^3.0^. A CCyR was defined either by the absence of Ph+ metaphases out of at least 20 metaphases or by <1% BCR-ABL1+ nuclei out of >200 nuclei. The molecular response was evaluated every 3 months and was reported according to the International Scale by reverse transcriptase-PCR of peripheral blood leukocytes.^34, 35, 36^ The tests were performed at one GIMEMA reference laboratory for 4 years, then also at other laboratories that had received their conversion factor through the EUTOS project^36^ and were certified by the Labnet GIMEMA network. A mutational analysis, by Sanger sequencing technique,^37^ was performed in the Bologna reference laboratory in all cases of loss of CCyR or MR^3.0^. The definition of the phases of the disease and of response was those recommended by EuropeanLeukemiaNet 2013.^3^

### Statistics

The Kaplan–Meier method^38^ was used to estimate overall survival. Death by any cause was the event of interest for overall survival. CCgR loss (CBA-positivity), MMR (MR^3.0^) loss and the probability of continuing INTERIM were calculated using the cumulative incidence procedure.^39^ Death was considered competing risk for CCgR and MMR loss, whereas death and refusal were the competing risks for the probability of continuing INTERIM.

## Results

**Figure 1:**
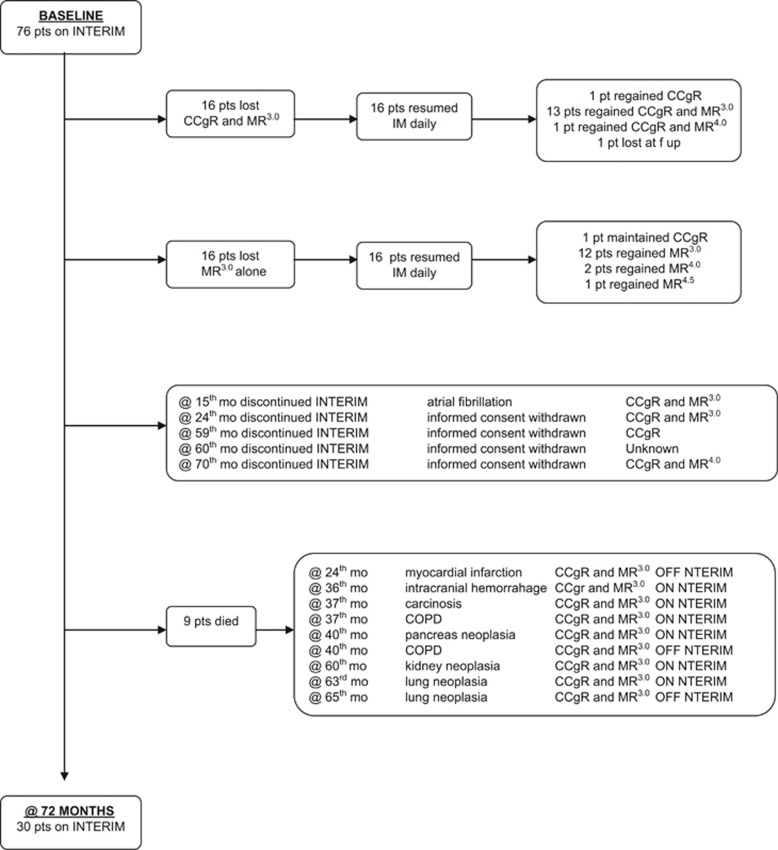
Flow diagram of INTERIM study—update at 72 months.

The results of intermittent imatinib treatment, with a median follow-up of 5.75 years (range 2.0–6.6 years) are shown in the flow diagram (Figure 1). Sixteen patients (21%) lost CCyR and MR^3.0^, 11 of them during the first 2 years and 5 later on. One of these patients was lost to follow-up. All the remaining 15 patients recovered a CCyR, with MR^3.0^ in 13 patients and MR^4.0^ in one. Sixteen patients (21%) lost MR^3.0^ after the second year. All these patients recovered an MR^3.0^, and two of them achieved a DMR (MR^4.0^ in one case and MR^4.5^ in one case). One patient went off the study because of an atrial fibrillation. He was back on standard imatinib, then was switched to nilotinib and is in MMR. Four patients withdrew their consent after 24–70 months. They went back to standard daily imatinib; 3 are in MR^3.0^, the fourth achieved a MR^4.0^ and is currently in TFR. Nine patients (12%) died after 24–60 months, being in MR^3.0^, of the causes that are listed in the flow diagram, namely another cancer (five patients), chronic pulmonary obstructive disease (two patients) and cardiovascular events (two patients). The median age at death was 75 years (range 72–80). No patients progressed to accelerated phase or blastic phase. No BCR-ABL1 kinase domain point mutations were detected at the time of CCyR or MR^3.0^ loss.

**Figure 2:**
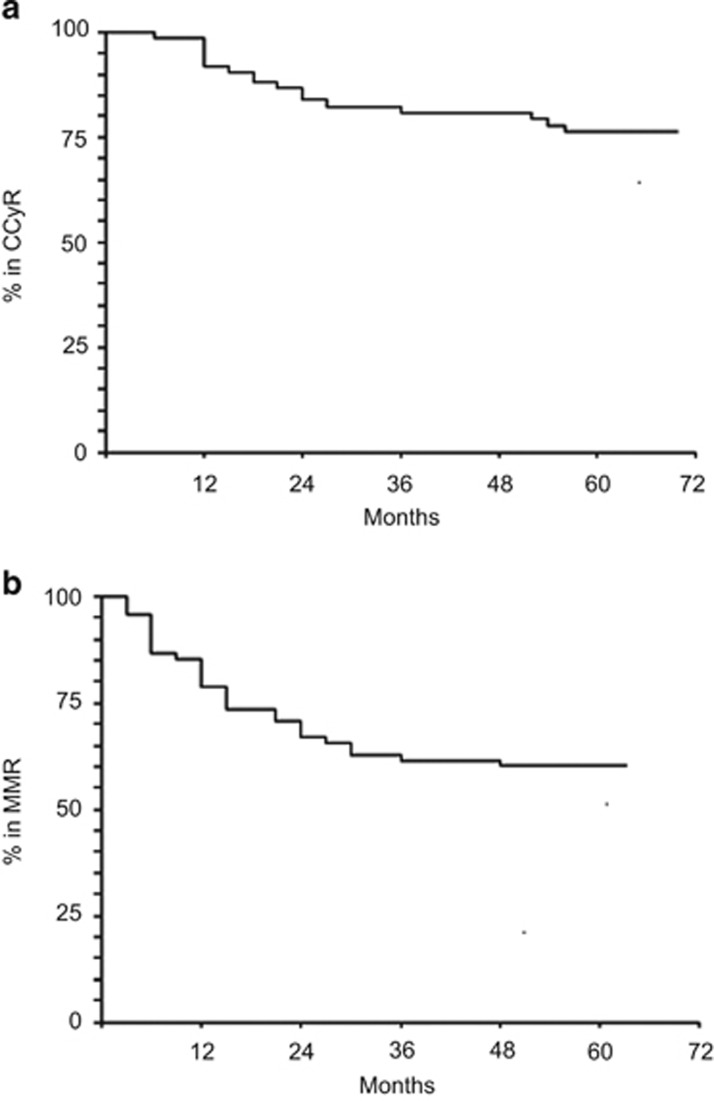
(**a**) Probability of maintaining the CCyR. In all, 16/76 patients (21%) lost CCyR, of whom 11 during the first 2 years and 5 later on. The probability of remaining in CCyR was 84% (95% CI 73–90) at 2 years, 81% (95% CI 69–88) at 4 years and 76% (95% CI 64–84) at 6 years. All 16 patients but 1 who was lost to follow-up were back to continuous imatinib treatment, same daily dose, and recovered the CCyR. (**b**) Probability of maintaining the MMR. In all, 32/76 patients (42%) lost MMR, including the 16 patients who had lost also the CCyR (Figure 1). The probability of remaining in MMR was 67% (95% CI 54–76) at 2 years and 60% (95% CI 47–70) at 4 and 6 years. All 32 patients but 1 who was lost to follow-up were back to continuous imatinib treatment, same daily dose, and recovered the MMR.

**Figure 3:**
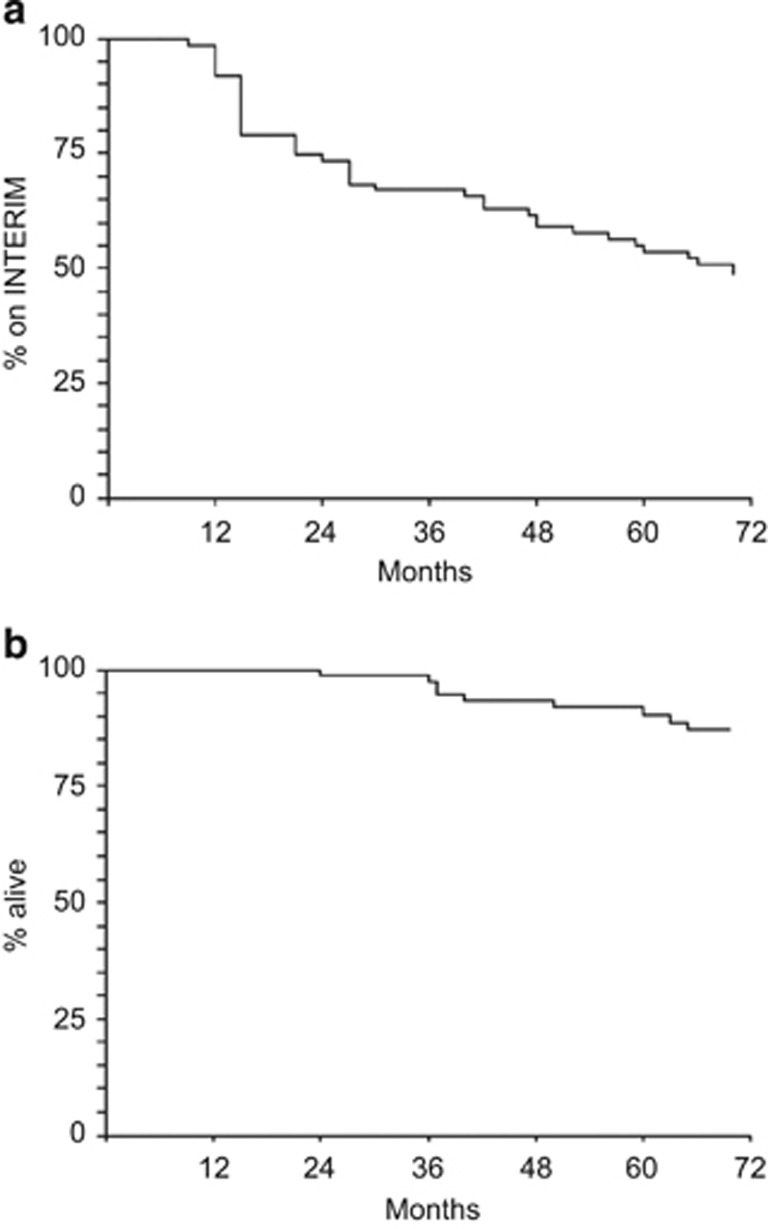
(**a**) Probability of remaining in the intermittent imatinib schedule (INTERIM). In all, 46/76 patients (61%) discontinued the intermittent schedule, of whom 24 during the first 2 years and 22 later on. The probability of maintaining the intermittent treatment schedule was 74% (95% CI 62–82) at 2 years, 59% (95% CI 46–69) at 4 years and 48% (95% CI 35–59) at 6 years. (**b**) Overall survival. No patients progressed and died of leukaemia. Nine patients (median age at death, 75 years) died in MMR for an overall survival of 87% (95% CI 78–95%) at 6 years.

The distribution over time of the loss of CCyR and of MR^3.0^ is shown in Figures 2a and b, respectively. The probability of maintaining the intermittent treatment schedule is shown in Figure 3a, where events were the return to the continuous, daily treatment, whatever the reason for that. At 6 years, 48% of patients were still on the intermittent schedule. Overall survival is shown in Figure 3b, where it should be noticed that all deaths occurred in remission. There were no progressions.

With a median follow-up of about 6 years, 30 patients are still on intermittent treatment taking the same imatinib dose as at baseline, 1 month on/1 month off. Four of them are in CCyR, 4 are in MR^3.0^, 20 are in MR^4.0^ and 2 are in MR^4.5^.

## Discussion

The current policies of TKI treatment of chronic phase CML mandate using TKIs at their respective approved or maximum tolerated doses lifelong, with the possibility of opening a window for treatment discontinuation when a DMR has been achieved and maintained for an as yet unspecified period of time.^3, 5, 12, 16, 40^ The window for treatment discontinuation can be enlarged in some of the patients who received imatinib first line, by switching early or late to a second-generation TKI,^40, 41, 42, 43^ as well as using second-generation TKIs first line.^17, 18, 19, 20, 44, 45^ Other policies have not been tested prospectively, particularly for treatments alternative to discontinuation, when discontinuation is not possible. The importance of the compliance to the treatment dose is highlighted by studies reporting that poor compliance is associated with a poorer molecular response.^46, 47^ However, it is time to open a debate not on compliance but on which dose should be used for chronic treatment, once a stable, optimal response has been achieved.

The concept of dose adaptation for chronic treatment can be tested in many different ways. Different schedules, such as a continuous daily treatment with a reduced dose or 1 day on/1 day off or 1 week on/1 week off, could be tested. In this exploratory study, it was decided to maintain the standard daily dose on a 1 month on/1 month off schedule. No pharmacokinetic studies were performed, but it is conceivable that the plasma concentration of imatinib fell to zero during the month off.

This study shows that a policy of imatinib reduction to 50% of the initial standard dose was associated with a substantial loss of response in 42% of patients but had no negative effects on outcome, particularly on progression and leukaemia-related deaths. It should be noted that at baseline all patients were in MMR, while after 6 years of intermittent treatment 22 of the original 76 patients (29%) were in DMR and could be eligible for a trial of treatment discontinuation. Therefore, even an intermittent schedule can improve the response, with time. A systematic, prospective study of the quality of life was not designed and performed. All these patients were tolerating imatinib well. Only 20 of them were complaining of minor side effects. In all, 50% of them reported the disappearance of the side effects, particularly of muscle pain and cramps, and of fluid retention. No evidence of a ‘discontinuation syndrome' was found, as it was reported in patients discontinuing imatinib in the EUROSKI study.^48, 49^ In this exploratory study, only elderly patients (⩾65 years) were selected and enrolled, because elderly patients tolerate TKIs less well, have more comorbidities, take more medications and have a shorter life expectancy. Moreover, the median age at diagnosis is already close to 60 years,^50^ and the proportion of elderly patients is destined to grow with time. However, also the younger patients who will not achieve a TFR will deserve attention. Although there is only another study (DESTINY study—ClinicalTrials.gov NCT01804985) looking for the minimum effective dose of any TKI, there is no doubt that the so called standard or approved dose is critical for achieving an optimal response as fast as possible and to prevent progression to blastic phase.^3, 5^ However, the issue is not to challenge the choice of the initial dose but to understand if the same dose is required indefinitely, and if so, for which purpose. This challenge has biological, clinical and financial implications. Biologically, almost all studies suggest that once an optimal response is achieved, the residual Ph+ cells may not be completely BCR-ABL1 addicted, and are resistant to TKI inhibition,^51, 52^ so that it may be necessary to consider other approaches testing other drugs in trials where toxicity and safety may prevail.^53^ In any case, those residual Ph+ cells can hardly give rise to new resistant Ph+ clones, because late relapses are exceptional.^9^ Therefore, the probability of dying of leukaemia becomes so small that one must worry more of other diseases and of the risk of treatment-related complications, a risk that will never be equal to zero, and that is difficult to predict over a very long period of time.^3, 5, 22, 23, 24^ From a financial perspective, the indefinite continuation of the standard, approved dose will expand the cost of the treatment exponentially.^30^ These considerations are also a valid argument in favour of a policy of treatment discontinuation and TFR, a policy that is more radical and more appealing.^11, 12, 16^ However, the point is not only which policy is ‘better'. The point is to acknowledge that a policy of TFR cannot be always successful, because at least 50% of patients are estimated to never reach a TFR, even with the largest use of second-generation TKIs. For the patients who do not achieve a TFR, it is necessary to reconsider some current concepts of treatment and to begin to look for a ‘minimum effective therapy'.

On these bases, we continue to work on the intermittent schedule with a standard daily dose, and we are now testing a progressive increase of the off-treatment period, up to 1 month on/3 months off.
